# Natural Bioactive Compounds Useful in Clinical Management of Metabolic Syndrome

**DOI:** 10.3390/nu13020630

**Published:** 2021-02-16

**Authors:** Annalisa Noce, Manuela Di Lauro, Francesca Di Daniele, Anna Pietroboni Zaitseva, Giulia Marrone, Patrizia Borboni, Nicola Di Daniele

**Affiliations:** 1 UOC of Internal Medicine-Center of Hypertension and Nephrology Unit, Department of Systems Medicine, University of Rome Tor Vergata, Via Montpellier 1, 00133 Rome, Italy; dilauromanuela@gmail.com (M.D.L.); francesca.didaniele@gmail.com (F.D.D.); annapietroboni@icloud.com (A.P.Z.); didaniele@med.uniroma2.it (N.D.D.); 2 PhD School of Applied Medical, Surgical Sciences, University of Rome Tor Vergata, Via Montpellier 1, 00133 Rome, Italy; 3 Department of Systems Medicine, University of Rome Tor Vergata, Via Montpellier 1, 00133 Rome, Italy; patrizia.borboni@alice.it

**Keywords:** metabolic syndrome, natural bioactive compounds, arterial hypertension, diabetes mellitus, dyslipidemia, low-grade inflammatory state, functional foods

## Abstract

Metabolic syndrome (MetS) is a clinical manifestation characterized by a plethora of comorbidities, including hyperglycemia, abdominal obesity, arterial hypertension, and dyslipidemia. All MetS comorbidities participate to induce a low-grade inflammation state and oxidative stress, typical of this syndrome. MetS is related to an increased risk of cardiovascular diseases and early death, with an important impact on health-care costs. For its clinic management a poly-pharmaceutical therapy is often required, but this can cause side effects and reduce the patient’s compliance. For this reason, finding a valid and alternative therapeutic strategy, natural and free of side effects, could represent a useful tool in the fight the MetS. In this context, the use of functional foods, and the assumption of natural bioactive compounds (NBCs), could exert beneficial effects on body weight, blood pressure and glucose metabolism control, on endothelial damage, on the improvement of lipid profile, on the inflammatory state, and on oxidative stress. This review focuses on the possible beneficial role of NBCs in the prevention and in the clinical management of MetS and its comorbidities.

## 1. Introduction

In the last century, there has been a rampant increase in chronic degenerative non-communicable diseases (CDNCDs) incidence. This, both because of the enhancement in the average life span of the population, and because of the spread of risk factors related to unhealthy lifestyle like smoking, alcohol abuse, sedentary, and incorrect eating habits [1].

Among the most common CDNCDs, an important role for the enormous worldwide spread is played by the metabolic syndrome (MetS). MetS is a clinic syndrome characterized by a cluster of multiple comorbidities, including hyperglycemia, abdominal obesity, arterial hypertension (AH) and dyslipidemia, which together represent an important risk factor for the onset of cardiovascular (CV) diseases and early death [2], as well as having an important impact on health care costs [3]. MetS has been estimated to increase the risk of all-cause mortality by approximately 1.5 times, with respect to the general population [4]. It is easy to understand how the spread of the MetS goes hand in hand with the spread of its comorbidities. It is estimated that around ¼ of the world’s population is affected by MetS, over a billion people, and this incidence is expected to increase [5]. The prevalence of MetS appears to be higher in the USA and Europe than in Eastern countries, although its incidence is slowly increasing in the latter as well, becoming a global epidemic. Its prevalence seems to be related to gender, in fact it is more frequent in male subjects compared to female ones [5,6]. These huge numbers make us reflect on the importance of finding new strategies to counteract this rampant world problem.

In this context, it is important to underline how an improvement in eating habits towards healthier lifestyles can play a fundamental role in the prevention and clinical management of MetS. Mediterranean diet (MD), rich in polyphenols, represents a healthy dietary pattern, characterized by regular consumption of fruit and vegetables and extra virgin olive oil (EVOO) as the main plant-based fat source; numerous studies suggest, in fact, that it is associated with the risk reduction in CDNCDs onset, including the MetS [7,8]. A recent study compared different diets such as plant-based diets, low-carbohydrates, low-fat diet, and MD, underlining that the latter is the most effective nutritional strategy for the prevention and treatment of MetS [9].

Focusing on preventive and therapeutic methods that are based on the use of natural bioactive compounds (NBCs), free from side effects, they could represent a valid adjuvant treatment to reduce hospitalization and health costs, as well as to improve the quality of life of MetS patients.

## 2. Metabolic Syndrome

MetS is characterized by the concomitant presence of multiple CV risk factors, which represent simultaneously the causes and the effects of MetS [10].

In 1988, MetS was called “syndrome X”, namely a particular condition that included alterations in glucose and lipid metabolism and AH [11]. Subsequently, MetS was defined by various organizations, as reported in Table 1 [5]. According to the World Health Organization (WHO), in the late 1990s, for MetS diagnosis, it was necessary that the patient had hyperglycemia or insulin resistance (IR), with blood glucose (BG) values >110 mg/dl, and postprandial BG >140 mg/dl, accompanied by at least one of the following factors: high-density lipoprotein cholesterol (HDL-C) <35 mg/dl in men or <40 mg/dl in women; triglycerides (TG) >150 mg/dl; central obesity defined by waist to hip ratio (WHR) >0.90 in men or >0.85 in women, or body mass index (BMI) >30 kg/m^2^; blood pressure (BP) >140/90 mmHg [12]. Furthermore, at first, microalbuminuria was included among the MetS diagnostic criteria, but subsequently has been removed [5]. In 2001 the National Cholesterol Education Program (NCEP) Adult Treatment Panel III (ATP III) defined MetS as the co-presence of at least three of the following factors: BG >100 mg/dl or pharmacological therapy for hyperglycemia; HDL-C <40 mg/dl in men or <50 mg/dl in women or lipid-lowering therapy; TG >150 mg/dl or lipid-lowering therapy; waist circumference (WC) >102 cm in men or >88 cm in women; BP > 130/85 mmHg or antihypertensive therapy [13]. Afterwards, in 2005, the International Diabetes Foundation (IDF) defined new criteria for MetS diagnosis, including obesity (WC ≥ 94 cm in men and ≥80 cm in women) as a necessary criterion. In addition, at least two of the following factors must be present: BG > 100 mg/dL or diagnosis of type 2 diabetes mellitus (T2DM); HDL-C < 40 mg/dl in men or <50 mg/dl in women or lipid-lowering therapy; TG > 150 mg/dl or lipid-lowering therapy; BP > 130/85 mmHg or antihypertensive therapy [14].

The three definitions focus on different metabolic alterations while describing the same clinical condition. In fact, the definition reported by the WHO focuses mainly on T2DM and CV risk, defining hyperglycemia or IR as a necessary criterion for MetS diagnosis. The NCEP ATP III definition, on the other hand, does not focus on a main pathology but on the co-presence of multiple risk factors. At last, the IDF defines the presence of obesity as necessary, determined by WC, which is however variable in different ethnic groups [15]. In fact, WC ranges were subsequently defined based on the patient’s ethnicity: for Europeans, Canadians, and Americans from the USA, WC ≥ 102 cm in men and ≥88 cm in women; for the Mediterranean, Middle Eastern, and sub-Saharan African populations, WC ≥ 94 cm in men and ≥80 cm in women; for Asian and Central-South American populations, WC ≥ 90 cm in men and ≥80 cm in women [16,17].

**Figure 1:**
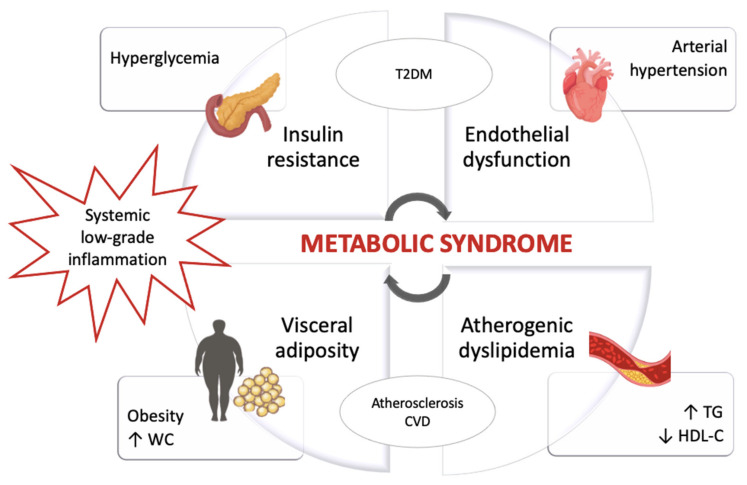
Pathophysiology of the metabolic syndrome. Abbreviations: ↑, Increase; ↓, Decrease; CVD, Cardiovascular disease; HDL-C, High-density lipoprotein cholesterol; T2DM, Type 2 diabetes mellitus; TG, Triglycerides; WC, Waist circumference.

The pathophysiological processes that involve the metabolic alterations typical of MetS are interrelated and characterized by a low-grade chronic inflammatory state [18], as shown in Figure 1.

A high accumulation of adipose tissue in the abdominal area represents a greater risk factor both for CV disease, for IR and T2DM [19,20]. In fact, the adipose tissue, divided into brown adipose tissue (BAT) and white adipose tissue (WAT), thanks to its endocrine role, regulates numerous metabolic pathways that, if altered, can lead to an impaired carbohydrate and lipid metabolism. BAT regulates adaptive thermogenesis [21] while WAT represents the storage site of lipids, releasing them in the form of energy when needed. Furthermore, WAT secretes leptin and adiponectin (important regulators of metabolic processes), adipsin, fatty acid-binding protein 4 (FABP4), fatty acid esters of hydroxyl fatty acids (FAHFAs) and palmitoleate; these substances act on the pancreas, liver, skeletal muscles, CV system, and brain. The WAT is in turn divided into visceral adipose tissue (VAT) and subcutaneous adipose tissue (SAT); only the former is associated with CV morbidity, as it determines a significant release in the blood of fatty acids and pro-inflammatory cytokines, like interleukin (IL)-6, IL-8 and tumor necrosis factor-α (TNF-α) [22,23], but also of plasminogen activator inhibitor-1 (PAI-1) capable to favor a prothrombotic state [24].

Excessive caloric intake causes hypertrophy and hyperplasia of the adipocytes, as occurs in obese subjects, resulting in metabolic alterations and in the onset of a low-grade chronic inflammatory state [25]. In fact, the pro-inflammatory substances that are released from adipose tissue can provoke IR, T2DM, metabolic, and CV diseases [25,26,27]. Some studies have identified in MetS patients, the presence of some inflammatory cytokines, such as IL-1β and IL-18, which play a crucial role in the development of atherosclerotic plaques [28,29,30].Atherogenic dyslipidemia is characterized by an increase in the level of circulating triglycerides, a reduction in HDL-C levels and therefore, an increase in low-density lipoprotein cholesterol (LDL-C). These alterations are closely related to impaired insulin metabolism. Indeed, in case of IR, it is possible to observe an increase in lipolysis and in apolipoprotein B (apoB) and very low-density lipoprotein (VLDL) levels, and a lipase dysfunction that leads to a reduction in the clearance of VLDL. Therefore, there will be a significant amount of TG-rich HDL lipoproteins, obtained from VLDL, which will be rapidly cleared by hepatic lipase, with a reduction in circulating HDL levels [10].

The chronic low-grade inflammatory state, the IR and the alteration of the lipid profile could lead to endothelial dysfunction [31]. Hyperglycemia can cause a glucose increase in endothelial cells, favoring the oxidative degradation of glucose metabolites, with a consequent oxidative stress (OS) [19]. Furthermore, they increase the levels of advanced glycation end products (AGEs) involved in the processes of vascular damage [32,33]. Another factor that induces the endothelial dysfunction in MetS patients is the insulin, which, in physiological conditions, could exert a vasodilator action; in fact, insulin acts on the phosphorylation of endothelial nitric oxide synthase (eNOS) which causes the nitric oxide (NO) increase, able to induce vasodilation. An alteration in the phosphorylation of eNOS, could lead to an enhancement of OS, inflammation, prothrombotic state and BP values [31,34,35].

## 3. Research Methods

The search of the paper (review or original articles) was conducted on Pubmed and Scopus electronic databases, until December 2020. The keywords used were: “metabolic syndrome” in combination with “natural bioactive compounds” and “body weight” or “body composition” and “metabolic alterations” and “endothelial dysfunction” and “lipid profile” and “inflammation” or “oxidative stress” or “gut microbiota modulation”. In addition, we included only papers in the English language. All the references were manually selected by the authors.

## 4. Natural Bioactive Compounds

Fruit and vegetables, and all plant-based products, are the key foods of the MD and, thanks to their polyphenols content, contribute to their well-known and well-studied cardioprotective and anticarcinogenic properties. These foods are rich in vitamins and minerals and contribute to achieve the right daily fiber intake. Moreover, they are rich in NBCs that induce numerous beneficial healthy effects [36,37]. NBCs are organic compounds of various derivations that exert important actions at the level of biological tissues. The NBCs of fruit, vegetables, and other plant-based foods are commonly called “phytochemicals” and can be classified into numerous categories based on their chemical structure and biological activity. Although it is hypothesized that many plant-derived bioactive compounds are yet to be discovered, more than 5000 phytochemicals have currently been isolated and studied [38]. These NBCs can be classified into phenolics, carotenoids, alkaloids, phytosterols, nitrogen-containing compounds, and organosulfur compounds [39]. Among these, the most studied are certainly the phenolics one. These are products of the secondary metabolism of plants, responsible for the typical coloring of fruit and vegetables, for the defense of the plant from microorganisms and other parasites, and commonly intervene in the growth, reproduction, and metabolism of the plant [40].

Numerous studies have highlighted their pivotal role in the onset and slowing progression of CDNCDs, such as CV diseases [41], cancer [39], diabetes mellitus [42], Alzheimer’s disease [43], and chronic kidney disease, both in the form of fresh food and as oral food supplements [44,45,46]. In fact, these NBCs seem to exert anti-inflammatory, antioxidant and antimicrobial effects and have cardioprotective, neuroprotective, hepatoprotective, hypoglycemic, and antihypertensive properties [47,48]. Precisely for this reason, the use of NBCs as adjuvant therapy could represent a valid therapeutic and preventive strategy to counteract the onset and the progression of numerous CDNCDs and of their comorbidities, such as the MetS.

## 5. Possible Effects of Natural Bioactive Compounds in Metabolic Syndrome

**Figure 2:**
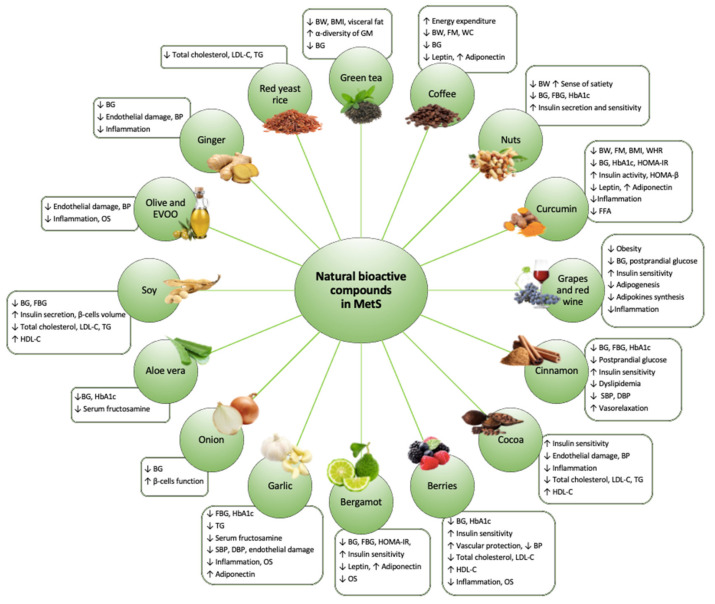
The effects of natural bioactive compounds on Metabolic syndrome management. Abbreviations: ↑, Increase; ↓, Decrease; BG, Blood glucose; BMI, Body mass index; BP, Blood pressure; BW, Body weight; DBP, Diastolic blood pressure; FBG, Fasting blood glucose; FFA, Fatty free acids; FM, Fat mass; GM, Gut microbiota; HbA1c, Hemoglobin A1c; HDL-C, High-density lipoprotein cholesterol; HOMA-IR, Homeostatic model assessment for insulin resistance; HOMA-β, Homeostatic model assessment for β -cell function; LDL-C, Low-density lipoprotein cholesterol; OS, oxidative stress; SBP, Systolic blood pressure; TG, Triglycerides; WC, Waist circumference, WHR, Waist to hip ratio.

Given the enormous worldwide spread of the MetS and all its clinical implications, it is extremely important to find a valid therapeutic strategy to counteract it. The first therapeutic approach applied in case of MetS is the modification of eating habits and lifestyle. It is in fact known how an improvement in eating habits, with a reduction in caloric intake in the case of overweight and obesity, a reduction in the intake of sodium, saturated fats, cholesterol, and simple sugars, can help in the clinical management of MetS comorbidities [49]. In some cases, it may be necessary to resort to pharmacological therapy. As there is not a single drug targeted against MetS, it is often necessary a poly-pharmaceutical therapy that can have side effects and reduce the patient compliance. For this reason, it is important to find a natural approach, free of side effects, which can help the MetS patients to delay the onset and progression of comorbidities typical of this syndrome [50]. Recently, numerous studies have highlighted how NBCs can play a crucial role in delaying the progression of MetS, exerting beneficial long-term effects (Figure 2).

### 5.1. Body Weight and Body Composition

The effect on body weight control and the reduction in visceral obesity exerted by NBCs has been demonstrated in numerous clinical studies (Table 2). Among the most studied NBCs for their potential anti-obesity action are catechins and their derivatives. In fact, these bioactive molecules seem to be able to reduce body weight through two main mechanisms: (I) increase in energy expenditure through stimulation of the sympathetic nervous system which, in turn, induces an enhancement in lipid oxidation, especially at the adipose tissue level [51]; and (II) reduction in intestinal absorption of lipids, contributing to the decrease in caloric intake [52]. Catechins are polyphenols belonging to the class of flavonoids, much studied for their antioxidant properties. Through their esterification, they can give rise to numerous molecules with bioactive properties, particularly detectable in green tea and cocoa.

Green tea is rich in epigallocatechin gallate (EGCG). Several clinical trials have shown that EGCG assumption is associated with a statistically significant reduction in body weight, BMI and visceral fat [53,54,55,56,69]. These effects have often been studied in association with the consumption of caffeine, which seems to act in synergy with EGCG in reducing body weight [70]. EGCG seems to be able to increase the activity of AMP-activated protein kinase (AMPK) which is involved in the reduction in fat biosynthesis and in the increase in fat catabolism, as well as in the improvement of insulin sensitivity, thus leading to a reduction in body weight [57,71]. Finally, EGCG would also appear to have a beneficial effect in the gut microbiota, increasing bacterial species such as *Bacteroides* and *Bifidobacterium*, improving the energy metabolism [72,73,74]. Pharmacokinetic studies have shown that the EGCG bioavailability, after oral administration, is dose-dependent. In fact, the pre-systemic elimination of tea polyphenols is saturable, consequently increasing their intake likely growths their bioavailability [58]. According to a recent study, the maximum plasma concentration of EGCG is obtained with the administration of EGCG extract after 8 h of overnight fasting, while the intake of EGCG as an oral food supplement reduces its intestinal absorption but improves the stability of the molecule in the body. Currently, in literature, there is no uniqueness about the EGCG dose needed to achieve the maximum beneficial effect [75].

Coffee is rich in NBCs, among these, the most studied are caffeine, belonging to the alkaloids, and chlorogenic acid, of the polyphenols class. In recent decades, the effect on the increase in energy expenditure, through the activation of thermogenesis, induced by caffeine, and its possible role in controlling body weight, has been much discussed. According to some studies, in fact, the habitual consumption of caffeine seems to be associated with the reduction in body weight, fat mass (FM) and WC [59,76,77,78]. The consumption of coffee is also able to prevent the accumulation of visceral fat and excessive body weight, thanks to the high content of chlorogenic acid, which seems to act thought the modulation of the peroxisome proliferator-activated receptor (PPAR) γ responsible of lipid metabolism [60,61].

Another food that acts on body weight control are nuts. Despite their high caloric density, the habitual consumption of nuts would seem to have an important effect on body weight control, especially if used as a snack instead of other foods rich in fats. Because these properties, the nuts are commonly used in diet-therapy for the body weight reduction [62]. Nuts are, in fact, functional foods rich in monounsaturated fatty acids (MUFAs), polyunsaturated fatty acids (PUFAs) as well as fiber, folate, calcium, magnesium, and potassium. Their action on body weight control would seem to be exerted by an increase in the sense of satiety [63,64,79,80]. Furthermore, the consumption of nuts has beneficial effects in the control of glucose and lipid metabolism, making them an excellent food for the MetS management [81].

Further studies focused on the role of curcumin, a natural phenol of *Curcuma longa*, in the modulation of obesity pathways. The consumption of curcumin, in association with a low-caloric diet, increases the loss of body weight, reducing FM%, BMI, and body circumferences [65,82,83]. In a recent in vitro study, Wu et al. highlighted that curcumin is able to inhibit the differentiation of adipocytes, and at the same time induces apoptosis of preadipocytes [66]. Curcumin also acts as an anti-inflammatory pathway, inducing the expression of adiponectin, a powerful anti-inflammatory agent produced by adipose tissue [67]. This results in an improvement in the chronic low-grade inflammatory status that occurs in obese subjects with MetS. Pharmacokinetic studies reveal that the bioavailability of curcumin after oral administration is very low, due both to poor intestinal absorption and to an important hepatic metabolism [84]. These obstacles can be overcome thanks to the use of specific inhibitors of the curcumin metabolism or the use of solid or liquid formulations that improve its absorption and slow down its elimination [85]. Furthermore, the effective safety of administering curcumin extracts has been demonstrated, in fact no toxic effects were observed up to the chronic daily intake of 180 mg of curcumin [86].

Quercetin, a flavonoid abundant in apples, grapes and red wine, seems to modulate in mitochondria the lipolysis pathways, inducing the body weight reduction [87,88]. Specifically, quercetin is able to decrease adipogenesis through the activation of AMPK [89] and the modulation of PPARγ [68]. Furthermore, quercetin seems to be able to reduce the synthesis of adipokines in adipose tissue, improving systemic low-grade inflammation. Therefore, quercetin would seem to act as a tissue-specific anti-inflammatory, contributing to the reduction in the expansion of adipose tissue induced by the inflammatory state [90]. It has been shown that the bioavailability of quercetin depends by the molecule carbohydrate fraction, in fact, its absorption is higher when it is assumed as glycoside rather than as free quercetin [91]. The food matrix in which quercetin is assumed, as a food or as oral food supplement, does not seem to influence the bioavailability of the flavonoid [92]. In addition, interindividual variability in the quercetin bioavailability was observed, possibly due to genetic polymorphisms, gut microbiota composition, BMI, and health status [93].

### 5.2. Metabolic Alterations

IR and T2DM play a crucial role in the pathogenesis of MetS. Therefore, numerous NBCs and their action on these pathological conditions have been analyzed over the years (Table 3) [50,94].

Among the NBCs, curcuma or turmeric appears to have an important modulatory function on T2DM and IR, indeed it has been studied for its anti-inflammatory, antioxidant and antidiabetic properties [95,143]. Some animal studies have highlighted the hypoglycemic role of curcumin and an improvement in insulin sensitivity following its assumption. Curcumin seems to play its hypoglycemic action through the inhibition of hepatic gluconeogenesis by an independent-insulin pathway. Furthermore, its antihyperglycemic role has been attributed both to its anti-inflammatory action thanks to the reduction in TNF-α and anti-lipolytic action with decrease in circulating levels of fatty free acids (FFAs) [96,97,98]. In particular, a study conducted on hamsters showed a significant reduction in homeostatic model assessment for insulin resistance (HOMA-IR) and in leptin levels, in animals treated for 10 days with curcumin compared to the control group [96]. Studies conducted on humans showed how turmeric appears to be able to reduce plasma glucose levels and improve insulin activity [99,100,101]. A study conducted on 240 prediabetic patients divided into a study group and in a control group, showed that none of the patients treated with curcumin (250 g/day for 9 months) developed T2DM, showing also a significantly higher homeostatic model assessment for β -cell function (HOMA-β) score [99]. Another research group treated overweight or obese diabetic patients with 300 mg/day of curcuminoids for 3 months; comparing the results obtained with the control group treated with placebo for the same period, the authors found a significant reduction in BG, hemoglobin A1c (HbA1c) and HOMA-IR in the study group [100]. Instead, Panahi et al. [101] used curcumin in association with piperine, an alkaloid contained in black pepper, on population of 100 T2DM patients divided equally into treated group and control group. The results obtained showed a significant reduction in circulating levels of glucose and HbA1c in patients treated with curcumin and piperine. The use of curcumin showed a reduction also in the leptin–adiponectin ratio, resulting from a decrease in the leptin levels and an increase in adiponectin levels. In fact, curcumin appears to be able to reduce the activity of the nuclear factor-κB (NF-kB) factor that is directly involved in inflammatory processes and in the synthesis of pro-inflammatory cytokines [144]. The reduction in inflammation results in an increase in adiponectin production and an inhibition of leptin synthesis [67,102]. The mechanism of curcumin action on these metabolic alterations typical of MetS, would seem to be related to its ability to reduce FFAs with a consequent decrease in BG. In fact, high circulating levels of FFAs are capable of causing IR as they are both associated with visceral fat deposits [97]. Furthermore, its strong anti-inflammatory activity prevents damage to pancreatic β cells, acting on NF-κB [95,145,146]. Indeed, a study conducted by Adibian M. et al. highlighted how the consumption of turmeric can counteract the complications of T2DM by reducing both TG and inflammatory biomarkers levels [95].

Cinnamon is a genus of the *Lauraceae* family from which is obtained the spice cinnamon, widely used since ancient times both in food and medicine [147]. Cinnamon can perform an action similar to insulin activating numerous mechanisms that lead to a reduction in BG [148]. The first human study about the beneficial effects of cinnamon on IR and T2DM was conducted by Khan A. et al., who showed a reduction in BG with three different doses of cinnamon *per* day (1, 3, and 6 g) in T2DM patients [103]. Numerous studies over the years investigated the compounds present in cinnamon and their beneficial effects on IR and T2DM. These healthy effects have often been associated with hydro-soluble polyphenols, more precisely with the flavonoids catechin and epicatechin [104]. A study conducted on MetS patients taking cinnamon hydro-soluble extract-based supplement for 12 weeks found a reduction in fasting blood glucose (FBG) [105]. The main action of hydro-soluble polyphenolic extracts seems to be a promotion of insulin signaling, resulting in increased IR-β insulin receptor and improved cellular glucose absorption. In fact, cinnamon promotes the increase in glucose transporter type 4 (GLUT4) acting as an insulin receptor that allows the entry of glucose into the cells [106]. Additionally, Jarvill-Taylor K.J. et al. [107] have highlighted how the hydroxychalcone present in cinnamon, induces a greater biosynthesis of glycogen, activating the glycogen synthase and inhibiting the glycogen synthase kinase3 β (GSK3 β). A clinical study conducted on healthy subjects evaluated the effect of a specific hydroalcoholic extract of Ceylon cinnamon on the reduction in post-prandial BG, showing a significant reduction in this parameter in the area under the curve (AUC) 0-60 min [108]. Furthermore, cinnamon seems to be a possible source of NBCs for the prevention and treatment of IR and T2DM, activating PPARγ and PPARα [109,149], that induce an improvement in dyslipidemia and increases the insulin sensitivity [150,151]. These actions of NBCs seem to improve, in combination with hypoglycemic therapy, the glucose metabolism of patients with IR and T2DM [110,149,152]. In fact, a study conducted by Crawford P. [110] found a reduction in HbA1c in T2DM patients treated with 1 g of cinnamon *per* day for 90 days in association with traditional hypoglycemic therapy, compared to the control group treated with hypoglycemic therapy alone. The study conducted by Sonal Gupta J. et al. [111] evaluated the effect of 3 g of cinnamon *per* day for 16 weeks on 116 MetS patients, comparing the results with a control group that took a placebo for the same period. At the end of the 16 weeks, numerous improvements were obtained in the treated group, regarding the lipid profile, body composition, FBG and HbA1c. Therefore, these results lead us to suppose that supplementation with cinnamon may lead to a reduction in the MetS risk factors and a decrease in its prevalence [111]. The cinnamon polyphenols bioavailability depends on several factors including the food matrix of assumption, the total polyphenols content and the gastrointestinal tract healthy state [153]. A study evaluated the effective bioavailability of polyphenols in a cinnamon-based drink, concluding that it is equal to 80% of the total content. The addition of sweeteners seems to increase this value. Furthermore, it has been observed that the polyphenols bioavailability is reduced when they assumed concomitantly with milk and its derivatives, as the polyphenols have a high affinity with the milk caseins [154].

Among the food rich in polyphenols, which exert a positive action on IR and T2DM, there are cocoa, coffee, and tea. Cocoa and its derivatives are natural products rich in catechins and flavonoids with numerous biological activities. Cocoa and dark chocolate contain a particular flavanols called flavan-3-ols, which appear to have a beneficial effect on CV disease, protecting against endothelial damage and improving insulin sensitivity [94,112,155]. Specifically, the study conducted by Grassi D. et al. [113] highlighted how the consumption of flavanol-rich dark chocolate (100 g *per* day for 15 days) was able to reduce IR and increase insulin sensitivity by improving pancreatic β cells functionality. The potential hypoglycemic role of cocoa depends also on its fiber content. In fact, the study conducted by Sarria B. et al. [156] detected a reduction in BG after daily consumption of soluble cocoa product rich in dietary fiber (10.17 g of total-dietary-fiber, 43.8 mg of flavanols and 168.6 mg methylxanthines *per* day) compared to the daily consumption of a product rich only in polyphenols (falavanols and methylxanthines with low fiber intake). Methylxanthines are a class of polyphenols with numerous health benefits. Among the methylxanthines, the cocoa theobromine seems to play a crucial role on glucose metabolism by regulating the intracellular levels of cyclic adenosine monophosphate (cAMP) which induces a greater release of insulin by the pancreas and glucose by the liver [157,158]. In addition, several studies have associated coffee methylxanthines with a reduced risk of developing T2DM [159,160]. Furthermore, caffeine has been associated with a dose-dependent reduction in glucose absorption [114,161,162]. Similar mechanisms have been highlighted in studies that evaluated the potential beneficial effects of tea on reduced risk of onset T2DM [163]. A study conducted on borderline MetS patients showed that the treatment based on green tea catechin extract reduced the risk factors of MetS in 68% of patients [115]. The hypoglycemic effect of tea was mainly associated with its catechin content, especially EGCG [117,118,164,165]. A study evaluated the effective bioavailability of caffeine and theobromine after methylxanthines assumption in the form of caffeine capsules, cola drinks, and chocolate. The authors observed that compared to the capsules, the absorption of caffeine was delayed when assumed in the form of food, while the absorption of theobromine from chocolate was faster [166]. Furthermore, it has been observed that the tissue distribution, the metabolism and the elimination of methylxanthines, such as caffeine and theobromine, can be altered by numerous factors such as obesity, smoking and alcohol abuse, sedentary lifestyle, and pharmacological therapy [167]. MetS is characterized by an increase in leptin levels and a decrease in adiponectin levels [168]. Several studies have demonstrated that coffee and tea had a modulatory action on these hormones, as well. Indeed, a recent meta-analysis has evaluated the effects of coffee consumption on circulating leptin and adiponectin levels, highlighting an inversely correlation between coffee consumption and leptin levels and a direct correlation with adiponectin [116]. Furthermore, a study evaluated the consumption of green coffee bean extract (400 mg *per* day) in association with a low-calorie diet, demonstrated a reduction in leptin and a significant increase in adiponectin in the treated group compared to the control group (placebo) [119]. A meta-analysis that instead examined the effect of green tea on leptin levels revealed that this hormone increased only in case of treatments lasting more than 12 weeks. Therefore, the anti-inflammatory effect of green tea, which involves a reduction in the synthesis of leptin, exercised only in case of prolonged treatment [120,169].

Berries (such as strawberries, blackberries, red fruits, blueberries, and raspberries) are functional foods rich in NBCs, including anthocyanins and flavonoids, with numerous biological properties [170]. Several studies have evaluated the hypoglycemic action of berries. Stull A.J. et al. [121] studied the effect of a smoothie, obtained from 22.5 g of blueberries rich in NBCs, consumed twice daily for 6 weeks by a group of obese patients with IR. At the end of the study, the treated patients showed an improvement in insulin sensitivity compared to the control group (placebo). Another study evaluated the metabolic effects of purified anthocyanins on 80 prediabetic and diabetic patients. The enrolled patients took 300 mg *per* day of purified anthocyanins obtained from bilberry and blackcurrant for 12 weeks. Treated patients showed a reduction in HbA1c compared to the control group (placebo), confirming a positive action of anthocyanins on glycemic control [122]. The anthocyanins in cranberry would seem to carry out their hypoglycemic activity as a consequence of the reduction in lipotoxicity. In fact, they act by activating the AMPK which leads an increase in GLUT4 transporters and glucose uptake, and the suppression of gluconeogenesis. In addition, AMPK acts on hepatic lipid metabolism, upregulating the gene expression of PPARα, acyl-coenzyme A (acyl-CoA) oxidase and carnitine palmitoyltransferase-1A [123,171]. Furthermore, anthocyanins seem to induce the secretion of incretin glucagon-like peptide-1 (GLP-1) that in turn, causes insulin secretion [171,172]. Among the berries, elderberry seems to exert important antioxidant, lipid-lowering and hypoglycemic roles in the treatment of T2DM and MetS patients. The patients treated with elderberry showed an improvement in HbA1c compared to no-treated subjects (control group) [124]. Elderflower extracts are able to activate PPARγ, stimulating the absorption of glucose [173]. Finally, the various berries seem to have different effects, based on their polyphenolic content and in the way of their consumption (for example fresh or frozen fruits) [174]. Pharmacokinetic studies suggest that berries anthocyanins are rapidly absorbed and eliminated, but their effective intestinal absorption rate has not yet been estimated [175]. In addition, anthocyanins appear to undergo important first-pass intestinal metabolic processes and circulate into the bloodstream as metabolites, which are the real effectors of the beneficial effects observed after berries assumption. Part of the anthocyanins assumed orally reach the colon at the level of which they may exert important effects on the gut microbiota [176].

Bergamot is a citrus fruit that has numerous health benefits. Bergamot essential oil contains bergapten and bergamottin with antioxidant, lipid-lowering and hypoglycemic action. Mainly, its hypoglycemic action was detected as a consequence of improvement of lipid metabolism. In fact, it has been shown that bergamot polyphenols are able to modulate lipid metabolism by inhibiting of hepatic 3-Hydroxy-3-Methylglutaryl-Coenzyme A (HMG-CoA) reductase and acyl-CoA cholesterol acyltransferase (ACAT) activities [8,125,177]. However, the bergamot polyphenols have also a direct hypoglycemic action by increasing the AMPK activity and stimulating glucose uptake in muscle and liver [126]. Furthermore, the polyphenols of bergamot act on the leptin–adiponectin ratio, increasing the levels of adiponectin and reducing those of leptin [127]. A study conducted to evaluate the action of bergamot extract (650, 1300 mg, and placebo) on MetS risk factors, showed an improvement in the lipid profile, a reduction in FBG, HOMA-IR, and insulin. In addition, the authors observed a reduction in leptin and ghrelin concentrations, and an increase in adiponectin. Therefore, supplements based on bergamot extract could be an effective in the treatment of the metabolic alterations typical of MetS [128].

Garlic is a spice of the *Alliaceae* family widely used since ancient times for its countless benefits. Some studies report its positive effects on the lipid profile and its hypoglycemic action [8]. A meta-analysis showed how garlic appears to reduce FBG and HbA1c levels in diabetic patients and those with CV diseases [178]. Its mechanism of action is linked to its ability to inhibit HMG-CoA reductase [129,130] and cholesteryl ester transfer protein (CETP) [131]. An improvement in the lipid profile leads to a decrease in BG and IR. A study, evaluating the effects of prolonged-release garlic powder tablets in T2DM patients, highlighted a reduction in the metabolic impairments. After 4 weeks, the patients treated with the tablets showed a better metabolic assessment, in particular the authors observed the reduction in TG, FBG, and serum fructosamine [132].

Onion is made up of numerous NBCs, including flavonoids, phenolic acids, quercetin, and others [8]. Among its many benefits, onion plays a positive role in reducing BG, allowing the physiological functions of pancreatic β cells through a mechanism involving in enhancement of skeletal muscle mitochondrial number and function [133,179].

Resveratrol, an abundant polyphenol in grapes and wine, exerts a positive effect in T2DM patients. The action of resveratrol in this patient’s population, is linked to the activation of AMPK and consequently of sirtuin1 (SIRT1) [134,135,180,181]. The latter activation induces an improvement of insulin sensitivity and glycemic homeostasis [182,183]. The flavonoic glycoside rutin, instead, present in various plant sources such as wine, peppermint, and citrus fruits, reduces the absorption of glucose in the small intestine. Indeed, it appears to be able to inhibit α-glucosidase, reducing the digestion of carbohydrates and thus decreasing postprandial BG [184]. In addition, rutin seems to stimulate insulin secretion, activate the translocation of GLUT4 and increase glucose uptake into tissues [136,185,186]. The bioavailability and metabolism of resveratrol are strongly dependent by the food matrix of assumption. A study compared the resveratrol bioavailability after assumption of grape juice, red wine, and oral food supplement based on resveratrol, highlighting how the bioavailability would appear to be greater after resveratrol assumption in natural and fresh products than an oral food supplement [187]. However, another study observed that the resveratrol absorption is strongly delayed after assumption of grape extracts allowing the molecule to be more metabolized in the intestine, increasing the concentrations of its active metabolites in the systemic circulation [188].

Aloe vera seems to have anti-diabetic properties. Some studies have investigated about aloe vera showing that it is able to reduce BG and fructosamine levels [94,137]. The meta-analysis conducted by Dick W.R. et al. demonstrated significant reductions in FBG and HbA1c [189]. The aloe vera assumption appears to be able to inhibit the glucose intestinal absorption, as well as stimulate the glucose catabolism and inhibit its synthesis [138]. Moreover, aloe vera has been shown to be able to increase the AMPK activity through the same metformin mechanism [139]. Therefore, the treatment with aloe vera of T2DM and MetS patients would seem to be an adjuvant tool to traditional pharmacological therapy [137,189].

Soy is known as a valuable source of proteins with high biological value, PUFAs and dietary fiber. In fact, soy is rich in phytochemical compounds, in particular isoflavones and phytosterols, which act with different, often synergistic, mechanisms that exert beneficial effects on human health [190]. Some studies report the antidiabetic effects of soy. A study conducted by Li et al., showed that black-soybean-leaf extract can exert hypoglycemic effects through the activation of hepatic AMPK and the PPARs pathways modulation [191]. Moreover, the estrogens contained in it seem to act on IR, improving insulin secretion and increasing the volume of pancreatic β cells [94,192]. Supplementation with soy isoflavones showed significant improvements in FBG and BG, leading to the hypothesis of their possible use as adjuvant treatment in T2DM and MetS patients [140,193]. The soy isoflavones bioavailability was investigated in pharmacokinetic studies. A high concentration of isoflavones, such as genistein and daidzein, was observed for several hours after ingestion of soy powder, suggesting that these compounds exhibit good bioavailability after its assumption [194]. Furthermore, soy isoflavones are more bioavailable when assumed in the glucoside form, more present in unfermented soybeans, compared to the aglycone form, whose concentration increases as result of the fermentation processes necessary to produce soy fermented derivatives [195].

Certain nuts categories have been shown to reduce the incidence of some CDNCDs, including T2DM. Hazelnuts are mainly composed of MUFAs and PUFAs, that exerts a cardioprotective role and they are rich in numerous NBCs [80,196,197]. Their consumption in T2DM and MetS patients seems to reduce BG and HbA1c, and improve insulin secretion and sensitivity thanks to their antioxidant, anti-inflammatory and lipid-lowering effects [8,198,199]. Walnuts and their tree components are also able to act positively on glycemic control [94]. Nuts α-Linolenic acid seems to modulate the circulating microRNAs involved in the control of insulin sensitivity [141]. Furthermore, the nuts complex set of MUFAs, PUFAs, and polyphenols can improve insulin sensitivity through the regulation of the phosphoinositide 3-kinase/protein kinase B (PI3K/AKT) signaling pathway [200]. A study evaluated the effect of the extract obtained from the leaves of the walnut plant (*Juglans regia* L.) in T2DM patients. The study population took two capsules (100 mg Juglans regia leaf per dose) for 3 months without interrupting their usual pharmacological therapy. At the end of 3 months, a significant reduction in FBG and HbA1c was highlighted, as well as an improvement in the lipid profile compared to the control group who took a placebo for the same time-period [142]. Therefore, the use of these products could support the usual hypoglycemic pharmacological treatments, improving the risk factors of MetS and delaying its onset.

### 5.3. Endothelial Dysfunction

Endothelial dysfunction is one of the complications of the MetS. The main causes of endothelial dysfunction are related to the metabolic alterations of T2DM, to dyslipidemia and to chronic low-grade inflammatory state [31]. Several NBCs seem to counteract and improve endothelial damage (Table 4) [201].

The NCBs present in garlic have been extensively studied for their hypotensive function. Indeed, garlic consumption would appear to induce a reduction in both systolic and diastolic BP [212,213,214]. One study evaluated the effects of garlic consumption on a population of MetS patients. They took 100 mg per kg body weight of raw crushed garlic, twice a day for 4 weeks, showing significant improvements not only in carbohydrates metabolism and in lipid profile, but also in BP. This study highlighted how an intake of garlic can prevent and improve MetS condition [215]. Garlic seems to be able to increase eNOS activity and to reduce the expression of vascular cell adhesion molecule 1 (VCAM-1), leading to a decrease in inflammation and OS, with a consequent protection by endothelial damage, as well as an improvement of BP values [202,216].

Cinnamon NBCs seem to modulate positively the MetS risk factors and exert an important antihypertensive action [148]. Various studies have evaluated this effect especially in prediabetic or T2DM patients, highlighting a reduction in BP and vasorelaxation [217]. Its mechanism of action seems to be related to the activation of chemosensory cation channel (TRPA1) capable to stimulate vasodilatory and vasorelaxant actions [203]. A study conducted on MetS patients, showed a significant reduction in BP after cinnamon assumption with consequent decrease in MetS prevalence compared to the control group [111]. A recent meta-analysis evaluating the effects of oral cinnamon supplementation on BP, confirmed its hypotensive effect on both systolic and diastolic BP [218].

Olive is rich in polyphenolic compounds, such as oleuropein, hydroxytyrosol and tyrosol with several beneficial actions. Olive tree leaf through the reduction in inflammation and OS, decrease endothelial damage [219]. EVOO, one of the basic foods of the MD, is associated with numerous health benefits. Several authors have associated consumption of EVOO with a reduction in BP [204,205,220,221,222,223]. The NBCs present in the EVOO seem to act by increasing of the eNOS expression and the NO synthesis [206,222]. Furthermore, it appears to be able to regulate the NF-κB and activator protein-1 (AP-1) activities [223,224]. Regular consumption of EVOO can lead to significant improvements in MetS patients, both in terms of BP control and in ameliorating other risk factors [7,225].

Ginger, from the Zingiberaceae family, has been shown to have hypoglycemic and hypotensive properties [226]. A recent meta-analysis highlighted that an intake of ginger (≥3 g *per* day for more than 8 weeks) was related to a significant reduction in AH [207]. Its beneficial action seems to be related to its ability to act on PPARs, AMPK, and NF-κB signaling pathways. Therefore, it allows a reduction in the inflammatory state and in the adipogenesis, increasing glucose uptake. These mechanisms prevent the onset of AH and endothelial damage [227]. It has been shown that the ginger phenols have a high stability in the gastric fluids. However, pharmacokinetic studies have demonstrated an important first-pass metabolism, which reduces the systemic concentration of active phenols [228]. In addition, the ginger phenols shown to have a wide distribution in various tissues, including the stomach, intestines, liver, lungs, and kidneys, which probably represent the main target organs [229].

Other foods rich in NBCs can play a protective role on endothelial damage. Among these, there is cocoa. In fact, it appears to be responsible for the increase in eNOS and NO, reducing endothelial damage and BP values [113,208,230]. Cocoa flavanol, thanks to its anti-inflammatory capacity, has a positive action on organ damage related to AH, improving endothelial function, allowing vasodilation, and inhibiting the platelets activation [230,231].

Foods containing resveratrol perform a protective vascular action [232]. Several studies report positive results of resveratrol on reducing BP by activating the cyclic guanosine monophosphate (cGMP)-dependent protein kinase 1α (PKG1 α) activity which exerts a vasodilatory-NO dependent action [209,210,211]. Therefore, its mechanism of vascular protection would seem to be related to its high antioxidant capacity, acting on all the risk factors of MetS [233].

### 5.4. Lipid Profile

Dyslipidemia is one of the main conditions that characterize the MetS. In fact, low HDL-C levels and increased blood TG levels are often observed in MetS patients. To improve the lipid profile of these patients, it is possible to resort to NBCs, capable of modulate the lipid metabolism (Table 5).

Resveratrol is able to act on lipid metabolism through numerous mechanisms, promoting the reduction in adipogenesis and the increase in lipolysis [241]. Specifically, the main mechanism of the SIRT1 pathway, a nicotinamide dinucleotide (NAD+)-dependent deacetylase, involved in energy and mitochondrial metabolism. Imamura et al. have shown that resveratrol is able to increase the expression of SIRT1, preventing the accumulation of TG in differentiated 3T3-L1 preadipocytes. The authors conclude that resveratrol regulates the expression of PPARγ, responsible of the lipid metabolism [234]. Analyzing the most recent clinical trials, resveratrol appears to reduce plasma concentrations of total cholesterol and TG [235] but it does not affect HDL-C and LDL-C concentrations [242].

Several clinical studies have shown an important action on the control of the lipid metabolism by cocoa and dark chocolate. Their assumption, from 2 to 12 weeks, would seem to be able to reduce plasma levels of total cholesterol and LDL-C [243]. Mursu et al. showed that daily consumption of 45 g of dark chocolate for 3 weeks seems to increase the serum HDL-C concentration in a group of healthy subjects [244]. In another study, Hamed et al. observed an improvement in the lipid profile, in particular a reduction in LDL-C and an increase in HDL-C, in healthy subjects taking 700 mg/day of flavonoids in the form of dark chocolate for one week [236]. More recently, a randomized and placebo-controlled study conducted by Leyva-Soto et al., revealed that total cholesterol, LDL-C and TG decreased after a daily intake of 2 g of dark chocolate (70%) for 6 months [237]. The cocoa polyphenols regulate the main pathways involved in both fat synthesis and catabolism. In particular, the main targets of cocoa polyphenols are NF-κB, AP-1, PPARs, and AMPK pathways [245]. All this evidence suggest that flavonoids are able to improve the lipid profile, if consumed daily. The bioavailability and metabolism of cocoa polyphenols has been extensively studied. Pharmacokinetic studies have highlighted their moderate bioavailability in a dose-dependent manner after intestinal absorption [246]. A study has shown that the absorption of cocoa polyphenols can be significantly increased with a concomitant intake of carbohydrates [247]. However, another study highlighted how the phase II metabolism of cocoa polyphenols appears to be increased by the presence of sucrose and milk in chocolate, suggesting a greater bioavailability of polyphenols in dark chocolate [248].

Red yeast rice is a food of Chinese origin, obtained from the fermentation of rice by *Monascus purpureus*. This treatment allows to red yeast rice to be rich in substances called monacolins. Among these, there is monacolin K, with well-known lipid-lowering properties, able to reduce total cholesterol and LDL-C [249]. Red yeast rice, in fact, seems to act in a similar way to statins, inhibiting HMG-CoA reductase, a key enzyme in cholesterol synthesis [238,250]. Thanks to this mechanism of action, it is called “natural statin”. Red yeast rice is also rich in pigments, organic acids, flavonoids, and natural phytosterols that contribute to the lipid-lowering action [251,252], namely reducing total cholesterol, LDL-C and TG. These results suggest that red yeast rice is an excellent oral food supplement without side effects, useful for the treatment of dyslipidemia, in particular in subjects intolerant to statins [253]. Pharmacokinetic studies propose that red yeast rice monacolin K exhibits poor bioavailability after oral administration, less than 5%. This is mainly due to an important intestinal and hepatic metabolism [251]. However, the monacolin K bioavailability would appear to be higher if assumed through the food matrix than as oral food supplement. This phenomenon is due to a higher dissolution rate which increases intestinal absorption [254].

Recent studies have shown that berries are able to improve the concentrations of total cholesterol, LDL-C and HDL-C, reducing CV risk in T2DM and MetS patients [170]. In particular, berries anthocyanins interact with the pathways of lipid metabolism, reducing the gene transcription responsible of fatty acids the synthesis stimulating their catabolism [170]. Furthermore, the berries anthocyanins would seem capable of inhibiting the CETP activity [239], improving lipid profile. The berry anthocyanins also have important antioxidant and anti-inflammatory effects, as well as lipid-lowering action, which help to protect against the main comorbidities of CV and metabolic diseases [255].

Soy is rich in isoflavones and phytosterols that had a beneficial role on lipid profile [190]. Specifically, numerous clinical studies demonstrated that soy isoflavones are able to decrease serum levels of total cholesterol, LDL-C and TG and showed slight effects on the increase in HDL-C, only after treatments longer than 12 weeks [240,256]. The authors also observed a reduction in LDL-C–HDL-C ratio [257]. In particular, soy isoflavones appear to be capable to modulate estrogen gene transcription. Specifically, soy isoflavones act through the activation of the PPARγ pathway, modulating positively the levels of circulating cholesterol [258].

### 5.5. Inflammation and Oxidative Stress

The presence of a low-grade chronic inflammatory state and an imbalance between pro- and antioxidant species that induce OS condition, are the basis of all the comorbidities of MetS. These conditions can be counteracted by taking NBCs, tested in in vitro studies and in numerous clinical trials (Table 6).

In recent years, EVOO has attracted particular scientific interest for its antioxidant and anti-inflammatory properties. EVOO has beneficial properties that counteract the onset and progression of CDNCDs [259,260,261,272,273]. These properties seem to be exerted by the antioxidant and anti-inflammatory action of the polyphenols that it contains, in particular hydroxytyrosol and oleocanthal. Hydroxytyrosol is a phenolic alcohol with important antioxidant actions. In fact, hydroxytyrosol seems to act as a free radical scavenger, donating hydrogen atoms to the peroxyl radicals [274]. At the same time, hydroxytyrosol would seem to implement the body’s endogenous defenses through various mechanisms, including the activation of the nuclear factor E2-related factor 2 (Nrf2), involved in the expression of phase II detoxification enzymes [262]. Furthermore, hydroxytyrosol appears to exert an anti-inflammatory action. Several in vitro and in vivo studies suggest that hydroxytyrosol is able to inhibit the synthesis of pro-inflammatory cytokines (like TNF-α) and the activity of cyclooxygenases (COX)-2 [263,264]. Pharmacokinetic studies have highlighted how the dose-dependent bioavailability of hydroxytyrosol is greater when assumed by EVOO compared to other food matrices such as margarine, wine, and pineapple juice. These results demonstrated how the oily nature of EVOO was important for the absorption of hydroxytyrosol [275]. The hydroxytyrosol bioavailability, like other phenols, is strongly influenced by factors such as age, gender, and hormonal order. In addition, hydroxytyrosol and its metabolites have an excellent distribution in all body tissues at the level of which it exerts its beneficial effects [276].

Oleocanthal is a secoiridoid responsible for the pungent taste of EVOO, which pharmacological mechanisms seem to counteract the inflammatory state. In fact, oleocanthal has an important anti-inflammatory action similar to that of ibuprofen (non-steroidal anti-inflammatory drug), as it is capable of inhibit COX-1 and COX-2 enzymes, and consequently the synthesis of inflammatory prostaglandins, in a dose-dependent manner [277]. Moreover, oleocanthal would seems to inhibit the synthesis of pro-inflammatory cytokines, such as TNF-α, IL-6, and IL-1β [265]. In a recent study conducted by Carpi et al., it was observed that oleocanthal is able to modulate the gene expression of genes related to inflammation in preadipocytes. In fact, the authors suggest that oleocanthal seems to attenuate the activation of NF-κB, a transcription factor that responds to inflammatory stimuli and which in turn regulates the production of cytokines and adipokines. This data proposes that oleocanthal plays a key role in the modulation of inflammatory processes at the level of adipose tissue and could therefore improve the chronic low-grade inflammatory state, typical of obesity-related diseases and MetS [266]. However, in vivo pharmacokinetic studies conducted in rats demonstrated a low intestinal absorption of oleocanthal [278]. Human studies investigating the oleocanthal bioavailability after EVOO assumption are limited. Only one study found good oleocanthal concentrations in human urine after 2–6 h from EVOO consumption [279]. Probably the oleocanthal bioavailability in humans is higher than in rats.

Quercetin has proven anti-inflammatory and antioxidant properties [280,281]. An in vivo study conducted on 60 subjects showed a statistically significant reduction in IL-6 and C-reactive protein (CRP), after a supplementation of 500 mg/day of quercetin for 8 weeks [267]. Furthermore, quercetin seems to be able to reduce COX-2 [268] and inducible nitric oxide synthase (iNOS) gene expression [269,282]. Quercetin can improve the body’s antioxidant defenses by stimulating the synthesis of glutathione (GSH) and modulating the expression of catalase, GSH peroxidase and superoxide dismutase (SOD) [281].

Supplementation with resveratrol could be a valid strategy for the improvement of the inflammatory state of MetS. Several studies suggest that resveratrol is able to reduce the production of pro-inflammatory cytokines, decreasing the chronic low-grade inflammatory state. Resveratrol also exerts an antioxidant action, neutralizing reacting oxygen species (ROS) and reactive nitrogen species (RNS) [270,283].

Other NBCs useful for counteract the chronic low-grade inflammation, are allicin and its derivatives contained in garlic. Garlic compounds seems to inhibit the gene transcription of numerous pro-inflammatory cytokines, including IL-6, TNF-α, and IL-1β [284]. Furthermore, garlic shown an improvement of adiponectin in plasma levels in MetS patients [271]. A study evaluated the allicin bioavailability after assumption of several food matrices, observing a good allicin bioavailability (about 80%) after garlic powder capsules assumption and a very similar bioavailability after raw garlic assumption. The allicin bioavailability from garlic-based food assumption is equally good, ranging from 16 to 66%. This bioavailability was reduced when allicin was assumed after a protein-rich meal [285].

### 5.6. Effects of Natural Bioactive Compounds on Gut Microbiota Metabolites

The gut microbiota is considered an “ecological community” of bacteria localized in the intestinal tract, capable of maintaining the homeostasis in human body. The main activities, exerted by gut microbiota, are represented by extraction, synthesis, and absorption of nutrients and metabolites like vitamins, short-chain fatty acids (SCFA), etc. [286]. Regarding its composition, gut microbiota is constituted up to 90% by two phyla, namely by Firmicutes and Bacteroidetes [287]. The first one is characteristic of high-fiber and high-carbohydrate diets, while the second one is characteristic of a high-fiber and animal proteins foods diet [288]. Gut microbiota changes in the lifespan and multiple factors are able to interfere with its composition as dietary habits, aging, physical exercise, stress, drugs, etc. [289,290]. Moreover, numerous studies have shown that in presence of metabolic alterations like MetS, the gut microbiota composition results altered [291,292].

The main compounds of natural origin that “interact” with gut microbiota are represented by polyphenols [293,294,295,296]. Polyphenols, once introduced into the human body, are metabolized as xenobiotics, in fact their bioavailability is lower compared to micro- and macro-nutrients [297]. Several studies speculate that phenolic compounds, as well as their aromatic metabolites, are able to modulate the gut microbiota composition, exerting prebiotic effects and antimicrobial actions to counteract pathogens [298,299]. In particular, polyphenols thanks to the colonic microflora are transformed into bioactive compounds and studies both in vitro and in vivo have demonstrated that they exert a modulatory action in the composition of the gut microflora, inhibiting and promoting the release of compounds that modulate its composition [300]. For example, in vitro study conducted by Monagas et al. [301] on lipopolysaccharide-induced peripheral blood mononuclear cells of healthy subjects, revealed that dihydroxylated phenolic acids, a proanthocyanidins metabolite, induced an important anti-inflammatory action through the inhibition of pro-inflammatory cytokines like TNF- α, IL-1β, and IL-6. In a following study conducted by Rastmanesh [302], the author demonstrated that, in obese subjects, drinks like green tea, vinegar and foods like fruit, favor body weight reduction thanks to their polyphenols content, that modulates the gut microbiota both for the greater glycans-degradation of Bacteroidetes, and for the metabolites-derived production from polyphenols. Another study conducted by Zanzer at el. [303] demonstrated that polyphenols contained in spices are able to inhibit glucose uptake in the gut microflora. In particular, the authors highlighted that cinnamon and turmeric lowered plasma glucose up to 45 min, compared to controls. Moreover, turmeric, thanks to release-induction of peptide YY (PYY) diminished hunger, compared to the control group. The authors concluded that modulating glucose response and appetite, some spices should play an “adjuvant” role in the prevention of metabolic diseases.

A further action is due to the SCFAs release. The latter are particularly important because they act as modulators of colonic functions and of inflammatory and metabolic processes [304]. A recent in vitro study by Vamanu et al. [305], demonstrated that *Curcuma longa* extract, particularly rich in curcumin, changes the gut microflora composition of hypertensive patients through the increased release of SCFAs thus, in turn, enhances the *Enterobacteriaceae* and decreases *Bacteroidetes-Prevotella-Porphyromonas* genera. Several studies speculate that the modulation of metabolic pattern producing SCFAs may represent an innovative approach to counteract pathological conditions, such as MetS, chronic kidney disease, and AH, characterized by a low-grade inflammatory status [44,45,49,74,261,306]. In this perspective, the most SCFA studied is represented by butyric acid. Some activities ascribable to this compound are represented by the control of the inflammatory proliferation and of intestinal bacterial species growth (favorable to the body homeostasis) and by the reduction in OS [307].

The role exerted by NBCs, such as polyphenols, is attracting increasingly attention. In fact, the conscious use of NBCs on specific metabolic patterns could help the clinical management of CDNCDs, such as the MetS.

## 6. Conclusions

In conclusion, there is a clear evidence on the NBCs positive role in the clinical management of MetS and its comorbidities. In fact, their assumption, especially in the long term, presents numerous beneficial effects such as body weight control, glucose and lipid metabolisms improvements, BP control, endothelial protection, and finally reduction in chronic low-grade inflammatory state and OS. Therefore, this evidence suggests that the habitual assumption of functional foods rich in NBCs could represent a valid preventive strategy for MetS and it could be an adjuvant therapy in its treatment. However, even if the possible beneficial effects of a large number of NBCs have already been widely studied, further clinical trials on larger samples are needed, in order to understand their specific mechanisms of action useful in control of metabolic pathways. As future perspective, it is necessary to define international guidelines for determine the NBCs minimum effective dose and their duration of assumption for obtain the beneficial effects described in the literature.

## Figures and Tables

**Table 1:** Principal definitions of MetS.

	WHO [12] (Required Plus Other Two Conditions)	NCEP ATP III [13] (Three Conditions)	IDF [14] (Required Plus Other Two Conditions)
Glucose metabolism	Glycemia >110 mg/dl or IR (required)	Glycemia >100 mg/dl or hyperglycemia treatment	Glycemia >100 mg/dl or T2DM
Lipid metabolism	HDL-C <35 mg/dl (men) or HDL- C <40 mg/dl (women)	HDL-C <40 mg/dl (men) or HDL-C <50 mg/dl (women) or lipid-lowering therapy	HDL-C <40 mg/dl (men) or HDL-C <50 mg/dl (women) or lipid-lowering therapy
	TG >150 mg/dl	TG >150 mg/dl or lipid-lowering therapy	TG >150 mg/dl or lipid-lowering therapy
Obesity	WHR >0,90 (men) or >0,85 (women) or BMI >30 kg/m^2^	WC >102 (men) or >88 (women)	WC >94 (men) or >80 (women) (required)
Arterial hypertension	BP >140/90 mmHg	BP >130/85 mmHg or antihypertensive therapy	BP >130/85 mmHg or antihypertensive therapy
Other criteria	Microalbuminuria (removed)		

Abbreviations: ATP III, Adult Treatment Panel III; BMI, Body mass index; BP, Blood pressure; HDL-C, High-density lipoprotein cholesterol; IDF, International Diabetes Foundation; IR, Insulin resistance; MetS, Metabolic syndrome; NCEP, National Cholesterol Education Program; T2DM, Type 2 diabetes mellitus; TG, Triglycerides; WC, Waist circumference; WHO, World Health Organization; WHR, Waist to hip ratio.

**Table 2:** NBCs in body weight and body composition management.

NBCs	Studies	Pathways	Beneficial Effects
Green tea (EGCG)	[53,54,55,56,57]	↑ AMPK activity	↓ BW, BMI, visceral fat
Coffee	[58,59,60]	Modulation of PPARγ ↑ Energy expenditure	↓ BW, FM, WC
Nuts	[61,62,63,64]	↑ Vagal signal of fullness Slowing of intestinal emptying	↑ Sense of satiety ↓ BW
Curcumin	[65,66]	↓ Adipocytes differentiation ↑ Preadipocytes apoptosis	↓ BW, FM, BMI, WHR
	[67]	↓ NF-kB activity	↓ Inflammation
Quercetin	[68]	↓ Adipogenesis ↑ AMPK activity Modulation of PPARγ	↓ Obesity

Abbreviations: ↑, Increase; ↓, Decrease; AMPK, AMP-activated protein kinase; BMI, Body mass index; BW, Body weight; EGCG, Epigallocatechin gallate; FM, Fat mass; GM, Gut microbiota; NF-kB, nuclear factor-κB; PPAR, Peroxisome proliferator-activated receptor; SCFA, Short-chain fatty acids; WC, Waist circumference; WHR, Waist to hip ration.

**Table 3:** NBCs in metabolic alterations management.

NBCs	Studies	Pathways	Beneficial Effects
Curcumin	[95,96,97,98,99,100,101]	↓ glucose-6-phosphatase activity ↓ phosphoenolpyruvate carboxykinase activity ↑ AMPK activity ↓ NF-kB activity	↓ BG, HbA1c, HOMA-IR Improvement insulin metabolism, HOMA-β ↑
	[67,102]	↓ NF-kB activity	↓ Leptin, ↑ Adiponectin
Cinnamon	[103,104,105,106,107,108,109,110]	↑ IR-β insulin receptor ↑ GLUT4 ↓ GSK3 β Activating PPARγ and PPARα	↓ BG, FBG, HbA1c ↓ Postprandial glucose ↑ Insulin sensitivity
Cocoa	[111,112,113]	cAMP level modulation	↑ Insulin sensitivity
Coffee	[114,115]	↓ Glucose absorption	↓ BG
	[116]	↓ NF-kB activity	↓ Leptin, ↑Adiponectin
Tea	[117,118]	↓ Glucose absorption	↓ BG
	[119,120]	↓ NF-kB activity	↓ Leptin
Berries	[121,122,123,124]	↑ AMPK activity ↑ GLUT4 ↓ Gluconeogenesis PPARα activation Acyl-CoA oxidase and carnitine palmitoyltransferase-1A modulation ↑ GLP-1	↓ BG, HbA1c ↑ Insulin sensitivity
Bergamot	[125,126,127,128]	↓ HMG-COA reductase ↓ ACAT activity ↑ AMPK activity ↑ glucose cell uptake	↓ BG, FBG, HOMA-IR, ↑ Insulin sensitivity ↓ Leptin, ↑ Adiponectin
Garlic	[129,130,131,132]	↓ HMG-CoA reductase ↓ CETP	↓ FBG, HbA1c ↓ Serum fructosamine
Onion	[133]	↑ skeletal muscle mitochondrial number and function	↓ BG
Resveratrol	[134,135,136]	↑ AMPK activity Activation of SIRT1 ↓ α-glucosidase ↑ GLUT4	↓ BG, postprandial glucose ↑ Insulin sensitivity
Aloe vera	[137,138,139]	↓ glucose intestinal absorption ↑ AMPK activity	↓BG, HbA1c ↓ Serum fructosamine
Soy	[140]	↑ AMPK activity Modulation of PPAR pathways	↓ BG, FBG ↑ Insulin secretion, β-cells volume
Nuts	[141,142]	Circulating microRNAs regulation PI3K/AKT regulation	↓ BG, FBG, HbA1c ↑ Insulin sensitivity

Abbreviations: ↑, Increase; ↓, Decrease; Acyl-CoA, Acyl-coenzyme A; ACAT, acyl-CoA cholesterol acyltransferase; AMPK, AMP-activated protein kinase; BG, Blood glucose; CETP, Cholesteryl ester transfer protein; FBG, Fasting blood glucose; FFAs, Fatty free acids; GLP-1, Glucagon-like peptide-1; GLUT4, Glucose transporter type 4; GSK3 β, Glycogen synthase kinase3 β; HbA1c, Hemoglobin A1c; HMG-CoA, 3-Hydroxy-3-Methylglutaryl- Coenzyme A; HOMA-IR, Homeostatic model assessment for insulin resistance; HOMA-β, Homeostatic model assessment for β -cell function; IR, Insulin resistance; NF-kB, nuclear factor-κB; PPAR, Peroxisome proliferator-activated receptor; PI3K/AKT, phosphoinositide 3-kinase/ protein kinase B; SIRT1, sirtuin1; TNF-α, Tumor necrosis factor-α.

**Table 4:** NBCs in endothelial dysfunction management.

NBCs	Studies	Pathways	Beneficial Effects
Garlic	[202]	↑ eNOS activity ↓ VCAM-1 expression	↓ OS ↓ SBP and DBP endothelial damage protection
Cinnamon	[203]	TRPA1 activation	↓ SBP, DBP ↑ Vasorelaxation
Olive and EVOO	[204,205,206]	↑ eNOS activity ↑ NO synthesis NF-κB and AP-1 modulation	↓ OS ↓ Endothelial damage ↓ BP
Ginger	[207]	PPARα activation ↑ AMPK activity NF-κB modulation	↓ Endothelial damage ↓ BP
Cocoa	[208]	↑ eNOS activity ↑ NO synthesis	↓ OS ↓ Endothelial damage ↓ BP
Resveratrol	[209,210,211]	↑ PKG1 α activity	↓ OS ↓ BP

Abbreviations: ↑, Increase; ↓, Decrease; AMPK, AMP-activated protein kinase; AP-1, Activator protein 1; DBP, Diastolic blood pressure; NF-κB, Nuclear factor kappa-light-chain-enhancer of activated B cells; eNOS, Endothelial nitric oxide synthase; EVOO, extra virgin olive oil; PKG1, cGMP-dependent protein kinase 1α; PPAR, Peroxisome proliferator-activated receptor; SBP, Systolic blood pressure; TRPA1, Transient receptor potential ankyrin 1; VCAM-1, Vascular cell adhesion molecule 1.

**Table 5:** NBCs in lipid profile management.

NBCs	Studies	Pathways	Beneficial Effects
Resveratrol	[234,235]	↑ SIRT1 activity PPARγ modulation	↓ Adipogenesis ↓ Adipokines synthesis
Cocoa	[236,237]	PPARs pathways modulation ↑ AMPK activity ↓ NF-kB and AP-1 pathways	↓ Total cholesterol, LDL-C, TG ↑ HDL-C ↓ Inflammation
Red yeast rice	[238]	↓ HMG-CoA reductase	↓ Total cholesterol, LDL-C, TG
Berries	[239]	PPARs pathways modulation ↓ CETP activity	↓ Total cholesterol, LDL-C ↑ HDL-C
Soy	[240]	PPARγ modulation	↓ Total cholesterol, LDL-C, TG ↑ HDL-C

Abbreviations: ↑, Increase; ↓, Decrease; AMPK, AMP-activated protein kinase; AP-1, activator protein-1; CETP, cholesteryl ester transfer protein; HDL-C, High-density lipoprotein cholesterol; HMG-CoA, 3-Hydroxy-3-Methylglutaryl- Coenzyme A; iNOS, inducible nitric oxide synthase; LDL-C, Low-density lipoprotein cholesterol; NF-kB, nuclear factor-κB; PPAR, Peroxisome proliferator-activated receptor; TG, Triglycerides; SIRT1, sirtuin1.

**Table 6:** NBCs in inflammation and oxidative stress management.

NBCs	Studies	Pathways	Beneficial Effects
Hydroxytyrosol	[259,260,261,262,263,264]	Nrf2 activation ↓ TNF-α synthesis ↓ COX-2 activity	↓ Inflammation ↓ OS
Oleocanthal	[265,266]	↓ COX-1 and COX-2 activity ↓ IL-6 and IL-1β synthesis NF-κB modulation	↓ Inflammation ↓ OS
Quercetin	[267,268,269]	↓ COX-2 and iNOS ↑ GSH synthesis Catalase, GSH peroxidase and SOD activities modulation	↓ Inflammation
Resveratrol	[270]	↓ Pro-inflammatory cytokines synthesis	↓ Inflammation
Garlic	[271]	↓ TNF-α synthesis ↓ IL-6 and IL-1β synthesis	↓ Inflammation ↓ OS

Abbreviations: ↑, Increase; ↓, Decrease; COX, cyclooxygenases; EVOO, extra virgin olive oil; GSH, Glutathione; IL, Interleukin; NF-κB, Nuclear factor kappa-light-chain-enhancer of activated B cells; Nrf2, nuclear factor E2-related factor 2; RNS, reactive nitrogen species; ROS, reactive oxygen species; SOD, superoxide dismutase; TNF-α, Tumor necrosis factor-α.

## Abbreviations

Acyl- CoA	acyl- coenzyme A
ACAT	Acyl-CoA cholesterol acyltransferase
AGEs	Advanced glycation end products
AH	Arterial hypertension
AMPK ApoB	AMP-actived protein kinase Apolipoprotein B
AP-1	Activator protein-1
ATP III	Adult Treatment Panel III
AUC	Area under the curve
BAT	Brown adipose tissue
BMI	Body mass index
BG	Blood glucose
BP cAMP	Blood pressure Cyclic adenosine monophosphate
CDNCDs	Chronic degenerative non-communicable diseases
CETP cGMP	Cholesteryl ester transfer protein Cyclic guanosine monophosphate
COX	Cyclooxygenases
CRP	C-reactive protein
CV	Cardiovascular
EGCG	Epigallocatechin gallate
eNOS	Endothelial nitric oxide synthase
EVOO	Extra virgin olive oil
FABP4	Fatty acid-binding protein 4
FAHFAs	Fatty acid esters of hydroxy fatty acids
FBG	Fasting blood glucose
FFAs	Free fatty acids
FM	Fat mass
GLP-1	Glucagon-like peptide-1
GLUT4	Glucose transporter type 4
GSH	Glutathione
GSK3 β	Glycogen synthase kinase 3 β
HbA1c	Hemoglobin A1c
HDL-C	High-density lipoprotein cholesterol
HMG-CoA	3-hydroxy-3-methylglutaryl- coenzyme A
HOMA-IR	Homeostatic model assessment for insulin resistance
HOMA-β	Homeostatic model assessment for β -cell function
IDF	International Diabetes Foundation
IL	Interleukin
iNOS	Inducible nitric oxide synthase
IR	Insulin resistance
LDL-C	Low-density lipoprotein cholesterol
MD	Mediterranean diet
MetS	Metabolic syndrome
MUFA	Monounsaturated fatty acid
NAD+	Nicotinamide dinucleotide
NBCs	Natural bioactive compounds
NCEP	National Cholesterol Education Program
NF-κB	Nuclear factor-κB
NO	Nitric oxide
Nrf2	Nuclear factor E2-related factor 2
OS	Oxidative stress
PAI-1	Plasminogen activator inhibitor-1
PI3K/AKT	Phosphoinositide 3-kinase/ protein kinase B
PPAR	Peroxisome proliferator-activated receptor
PUFA	Polyunsaturated fatty acid
PYY	Peptide YY
RNS ROS	Reactive nitrogen species Reacting oxygen species
SAT	Subcutaneous adipose tissue
SCFAs	Short-chain fatty acids
SIRT1	Sirtuin1
SOD	Superoxide dismutase
T2DM	Type 2 diabetes mellitus
TG	Triglycerides
TNF-α	Tumor necrosis factor -α
TRPA1	Chemosensory cation channel
VAT	Visceral adipose tissue
VCAM-1	Vascular cell adhesion molecule 1
VLDL	Very low-density lipoprotein
WAT	White adipose tissue
WC	Waist circumference
WHO	World Health Organization
WHR	Waist to hip ratio
